# Monkeypox detection from skin lesion images using an amalgamation of CNN models aided with Beta function-based normalization scheme

**DOI:** 10.1371/journal.pone.0281815

**Published:** 2023-04-07

**Authors:** Rishav Pramanik, Bihan Banerjee, George Efimenko, Dmitrii Kaplun, Ram Sarkar

**Affiliations:** 1 Department of Computer Science and Engineering, Jadavpur University, Kolkata, West Bengal, India; 2 Department of Computer Science and Engineering, University Institute of Technology, Burdwan, India; 3 Department of Automation and Control Processes, Saint Petersburg Electrotechnical University “LETI”, Saint Petersburg, Russian Federation; Jeonbuk National University, KOREA, REPUBLIC OF

## Abstract

We have recently been witnessing that our society is starting to heal from the impacts of COVID-19. The economic, social and cultural impacts of a pandemic cannot be ignored and we should be properly equipped to deal with similar situations in future. Recently, Monkeypox has been concerning the international health community with its lethal impacts for a probable pandemic. In such situations, having appropriate protocols and methodologies to deal with the outbreak efficiently is of paramount interest to the world. Early diagnosis and treatment stand as the only viable option to tackle such problems. To this end, in this paper, we propose an ensemble learning-based framework to detect the presence of the Monkeypox virus from skin lesion images. We first consider three pre-trained base learners, namely Inception V3, Xception and DenseNet169 to fine-tune on a target Monkeypox dataset. Further, we extract probabilities from these deep models to feed into the ensemble framework. To combine the outcomes, we propose a Beta function-based normalization scheme of probabilities to learn an efficient aggregation of complementary information obtained from the base learners followed by the sum rule-based ensemble. The framework is extensively evaluated on a publicly available Monkeypox skin lesion dataset using a five-fold cross-validation setup to evaluate its effectiveness. The model achieves an average of 93.39%, 88.91%, 96.78% and 92.35% accuracy, precision, recall and F1 scores, respectively. The supporting source codes are presented in https://github.com/BihanBanerjee/MonkeyPox.

## Introduction

The present outbreak of the Monkeypox virus has had an adverse impact on the global health community. Monkeypox is a viral disease that can be transmitted from person to person (https://www.ecdc.europa.eu/en/news-events/epidemiological-update-monkeypox-outbreak). It was first diagnosed in 1970 [1], and since then the bulk of cases have been recorded in West Africa. The first case outside of Africa was recorded in the United States in 2003. Subsequently, other investigations have been carried out to determine the epidemiology of this virus. According to the World Health Organization (WHO), Monkeypox can be transmitted in two ways: from an animal to a human and from a human to a human. Data obtained recently by the European Centre for Disease Prevention and Control (ECDC) and WHO reveal that males accounted for 99% of cases in Europe (https://monkeypoxreport.ecdc.europa.eu/). The majority of occurrences are of men having sex with men (MSM) [2] in countries such as Canada, Spain and the United Kingdom. However, scientists also believe that the greater numbers of MSM are caused by close contact, rather the virus itself classified as a sexually transmitted disease [3]. According to the most recent accessible paperwork (https://www.who.int/news-room/fact-sheets/detail/monkeypox?gclid=Cj0KCQjwmdGYBhDRARIsABmSEeP5XAo6H7wvAszLbY2LGLgJHABSc_lcUb3zmD4GBxFaOIcBO6EqTEgaAmSwEALw_wcB), the community’s longest chain has expanded from 6 to 9. The Monkeypox fatality rate has recently been estimated to be 3-6%. According to the WHO, immunization against smallpox has been shown to be useful in the diagnosis of Monkeypox. It should be noted that Monkeypox is less infectious than smallpox. Its symptoms include fever, rashes, and enlarged lymph nodes.

The polymerase chain reaction (PCR) test is now considered one of the most efficient methods to diagnose Monkeypox (https://www.nist.gov/news-events/news/2022/07/nist-develops-genetic-material-validating-monkeypox-tests). Pox infections are frequently detected by the visual examination of skin lesions and rashes. Skin lesions and rashes caused by Monkeypox might seem similar to chickenpox and cowpox lesions and rashes. The clinical symptoms of Monkeypox are similar to those of smallpox but less severe in the case of Monkeypox. Due to clinical and visual similarities between Monkeypox diseases, it can be difficult for healthcare professionals to diagnose the early signs of Monkeypox. Computer-assisted diagnosis has gained popularity in recent years to assist medical practitioners in many difficult situations [4].

In the present work, we consider this as a classification task. Here, the system generally receives an image input, performs some processing, and then labels the image to a particular class as per the need. Nowadays, deep learning-based approaches are commonly used in the medical image processing domain due to their advantages over handcrafted feature extraction-based techniques [5]. Such approaches involve two components: feature extraction and classification based on the extracted features. Convolution operations are used to extract features, while multi-layered neural networks are applied to classify them. To achieve this objective, researchers frequently employ various forms of convolutional neural networks (CNNs). While CNNs have lately demonstrated significant generalization capabilities [6], the use of a single CNN model may not be adequate to address different and complex classification challenges.

Ensemble learning has also recently been the subject of extensive investigation [7]. Ensemble learning approaches seek to capture an association of accessible complementary information offered by base learners in order to make more correct predictions. Deep learners often provide a very high confidence score for both correctly and wrongly identified instances [8]. Thus, deep learning outputs (probabilities) can be processed effectively in order to capture an association of probabilities and create a robust prediction.

Aside from that the idea of transfer learning based models is commonly used to avoid the need for a substantial amount of data to develop competent CNN models. Initially, the network/model is trained on a very large dataset, and the trained weights are subsequently fine-tuned on a relatively small target dataset [9]. This act of transferring information from one domain to other benefits in reducing the reliance on huge amounts of training data for models to demonstrate high generalization properties.

To this end, we propose a Beta function-based ensemble network consisting of three base learners from diverse backgrounds. At first, we use some standard techniques to augment the data followed by online augmentation using Gaussian noise to further augment the training data. These samples are fed to three deep learners namely InceptionV3, Xception and DenseNet169 to generate probability scores. These scores are then normalized using the proposed Beta function-based normalization scheme. In the end, we use the sum rule-based aggregation for making the final class predictions. We extensively test our ensemble network on a publicly available dataset for Monkeypox detection using skin images. We use a 5-fold cross-validation scheme to ensure the robustness of the proposed model. In a nutshell, our contributions are listed below:

- We propose an ensemble of CNN models for Monkeypox detection using skin lesion images.
- We present a novel Beta function-based scheme for normalization of probability scores generated by the base CNN models.
- We evaluate our method on a publicly available skin lesion image dataset to test the effectiveness of the same.

The rest of the work is organized in the following manner: The related work section provides an overview of the recent works relating to medical image analysis using deep learning. In the methods and materials section, we first discuss the dataset used and then go into greater detail about the methodology. In the results and analysis section, we detail the result and attempt to analyse the same. Finally, we make some concluding remarks in the conclusion section and state some possible extensions of this work.

## Related work

In this section, we revisit some of the recent methods related to deep learning with its applications to medical image analysis.

Medical image analysis using deep learning techniques has seen a certain surge in recent times, owing to the easy availability of sophisticated hardware [10]. Typically, for a classification-based problem, more focus has been emphasised on the feature extraction part, which is probably the most essential part for any representation learning-based task. Recently the authors in [11] proposes a channel attention scheme for breast cancer classification. The authors aim to enhance the feature maps by the use of shuffling schemes between the channels of the feature maps. Oh et al. [12] use a patch-based strategy to train a ResNet-based CNN architecture which was trained with limited training data for COVID-19 detection. In an article by Zhang et al. [13], the authors proposed a one-class detection technique for the diagnosis of pneumonia. Specifically, the authors aimed to learn with the anomaly scores which they found of great significance. Further, an interesting work presented by Wang et al. [14] explores the possibility of self-supervised learning to train under constrained label circumstances. The authors used augmentation-based contrastive learning to perform the self-supervision-based pre-training task. Araújo et al. [15] proposes an augmentation technique based on patches for breast histopathology image classification.

Recently, there has been a considerable amount of research performed to extract deep features and select the most informative features and discard the redundant ones to form much better and separable decision boundaries. In a work by Basu et al. [16], the authors propose a deep feature selection approach for COVID-19 detection from Computed Tomography (CT) scans. In a similar work by Pramanik et al. [4], the authors propose a feature selection-based framework with a ResNet-50-based backbone. Cao et al. [17] present a patch-based attention network for cervical cancer detection using a DenseNet-169-based backbone network. The work by Shen et al. [18] proposes an end-to-end CNN model using region of interest information. There has also been progressing in research in developing CNNs with relatively low computational overhead. One such method was carefully designed in [19] for COVID-19 detection from Chest X-Rays.

The work by Khatami et al. [20] proposes wavelet transform-based deep belief networks for medical image analysis. The authors aim to capture an association of 3 models for medical image classification by utilizing the idea of capturing complementary information. The authors in [21] leverage a multi-scale ensemble approach to classify breast cancer images. In a recent work by Pramanik et al. [22], the authors use three transfer learning-based models with additional layers to learn data-specific features. Finally, the authors propose a novel fuzzy aggregation method which is based on the minimization of the observed and actual error values. In a separate study by Bhowal et al [23], the authors propose a game theory-based fuzzy integral for ensemble learning. This method was applied to breast cancer identification. Majorly the motivations of such methods lie in the fact that the aggregation method should capture the maximum possible complementary information.

### Literature interpretation

We observe the literature to have some brainstorming ideas for solutions to the challenges in the domain of medical image processing. In particular, there have been some preliminary studies performed in this domain [24–26]. However, deep learning-based architectures as we see them in the literature do not provide ultimate reliability most of the time [4, 16, 17]. Some of these methods are carefully modified for specific tasks [11, 19]. The majority of these methods are formulated to extract better feature maps in the feature extraction part. In particular, medical image processing is a sensitive topic, considering the fact that a wrong diagnosis is not at all acceptable. Specifically, diseases that are uncommon, such as Monkeypox, are affected due to a lack of proper diagnostic methods.

In these situations, development of a robust and reliable method plays a vital role. In the past, researchers have investigated several ensemble learning models [19, 22]. As stated earlier, the aim of an ensemble learning model is to maximize performance by aggregating decisions to provide a more reliable decision. Deep learners typically tend to provide high confidence scores for even incorrect classification scenarios [8]. Thus, when designing an ensemble learning model, this fact should be taken into account. In this work, to bridge this gap, we have provided an ensemble learning-based methodology to identify Monkeypox in skin lesion images.

## Materials and methods

**Fig 1:**
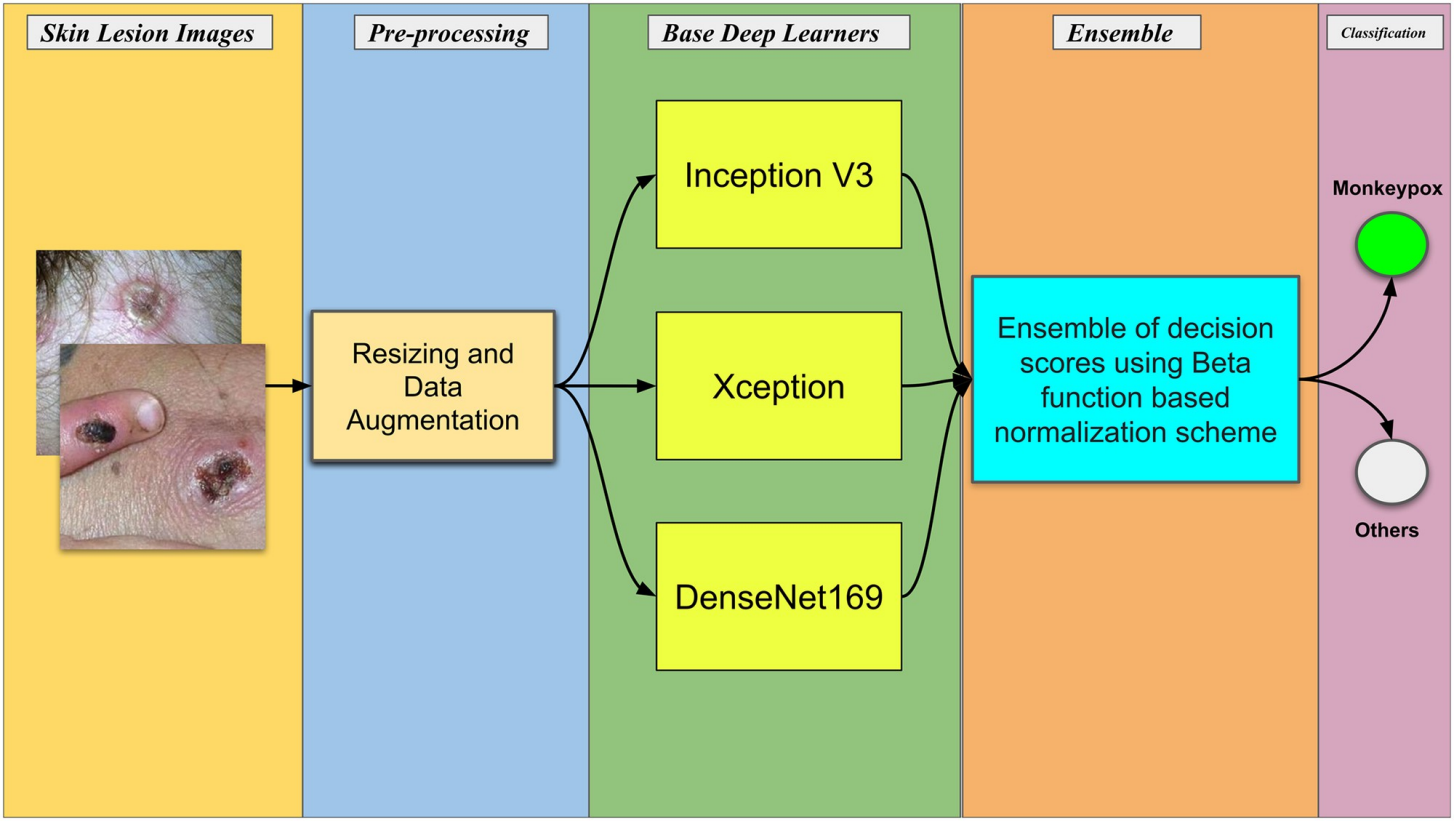
The overall pipeline of the present work for Monkeypox detection from skin lesion images.

In this section, we first discuss the dataset we have experimented on followed by introducing the proposed model for identifying Monkeypox from skin lesion images. We first resize the training samples to 224 × 224 pixels. Since we deal with a relatively small-sized dataset, we need to take care of a major challenge while training a CNN model, i.e., the problem of overfitting. To deal with this, we augment all the training images by utilizing augmentation techniques including horizontal and vertical shifting, brightness changing, zooming, channel shifting, horizontal and vertical flipping, rotating, and changing. Additionally, we consider color spaces like YUV and HSV to make sure our framework learns discriminative embeddings. Further, these training images are then fed to these three pre-trained (pre-trained on the ImageNet dataset) CNN models, namely Xception, InceptionV3 and DenseNet169. Before feeding the images, we further augment them using Gaussian noise. These pre-trained CNN models are fine-tuned using this target Monkeypox Skin Lesion dataset including its inner convolutional layers. Finally, to have a better decision over the predicted probability scores of the individual models, an enhancement scheme is proposed based on the aggregation of Beta-normalized output values of the respective models using the sum rule. The overall pipeline of the proposed work is presented in Fig 1

### Dataset description

For evaluating the proposed method we use a publicly available dataset, namely Monkeypox Skin Lesion dataset [27] which is hosted in the Kaggle platform https://www.kaggle.com/datasets/nafin59/Monkeypox-skin-lesion-dataset. We consider the original images and segregate the train and test sets. There are 228 photos in this dataset, 102 of which are of the “Monkeypox” class and the remaining 126 being of the “Others” class, which includes cases of other skin lesion-based diseases like chickenpox and measles that are not Monkeypox.

### Addition of Gaussian noise

Generally, a deep CNN model needs a significant amount of data for proper training of the model. Otherwise, the model would overfit the training data if it has experimented with small-sized datasets. In the present work, this issue becomes relevant as we experiment with a relatively less number of image samples. Therefore, in each iteration, we add Gaussian noise (with mean = 0 and variance = 0.01) to the input data and this in turn introduces variability in the learning process, thus reducing the possibility of overfitting [28].

### Inception V3

**Fig 2:**
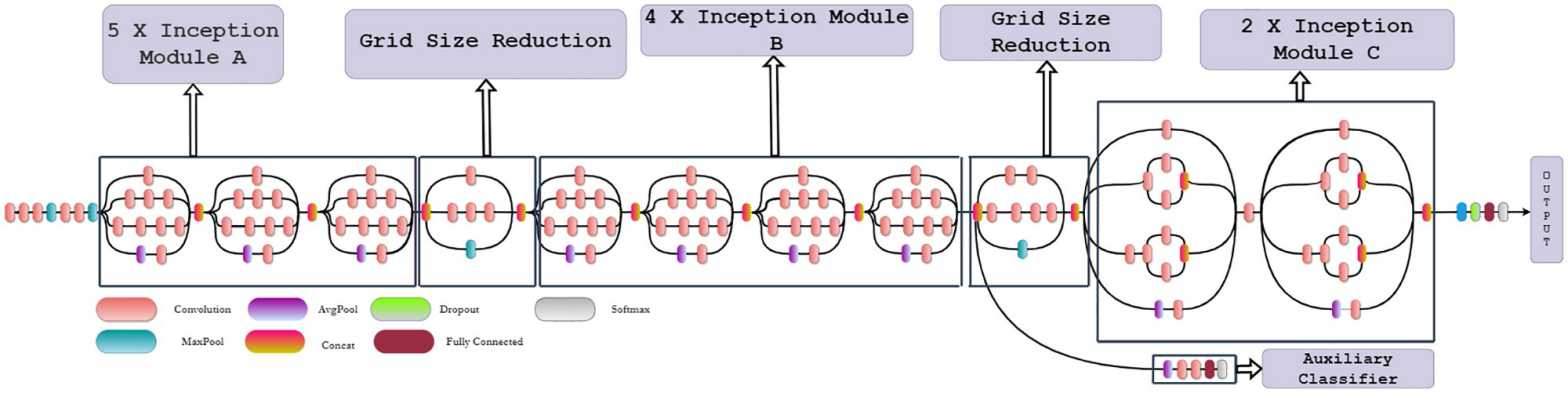
Architecture of inception V3. Modified from [29].

The family of InceptionNets focuses on training with low computational resources. Specifically, Inception V3 [29] uses asymmetric convolutions. The convolutions are factorized to help capture more diverse features using lower computational costs. Furthermore, with the aim of capturing an aggregation of these asymmetric features, these are concatenated before proceeding to the next layer. In addition, the use of an auxiliary classifier helps to counter the overfitting problem. In particular, the auxiliary classifier has also been used in the previous versions of the InceptionNets. The overview of the inception architecture is given in Fig 2

### Xception

**Fig 3:**
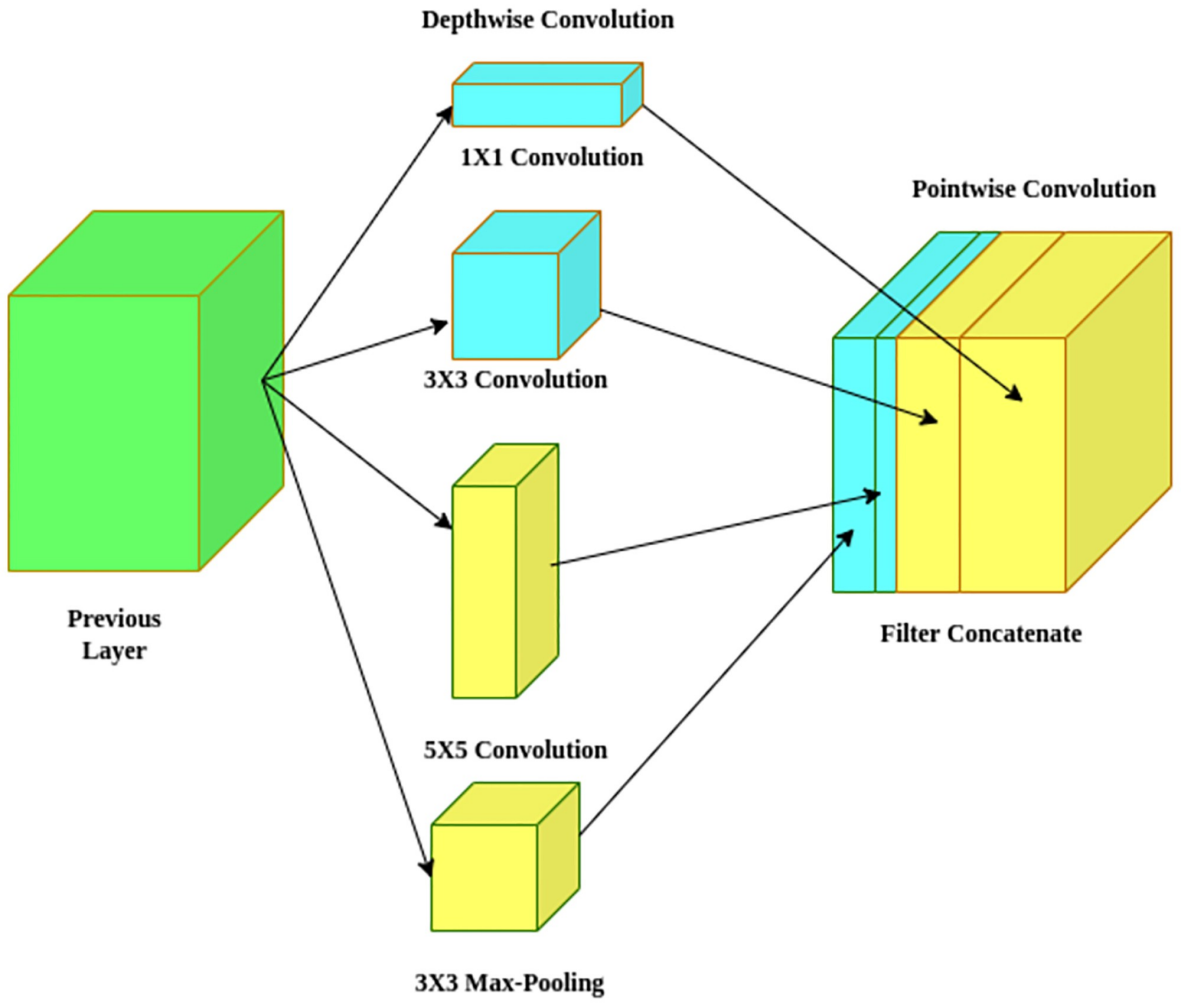
A basic block used of the Xception architecture.

Prior to leveraging 1x1 standard convolution across the depth to condense the input space, Xception [30] applies the filters independently to each depth feature map. This solution is almost analogous to a depthwise separable convolution that has been in use since 2014. One noticeable difference between Xception and other CNNs is that it does not introduce non-linearity with the rectified linear unit (ReLU). The author defends the idea that employing a nonlinear activation in a deeper network, similar to those in the Inception model, may be effective. However, information might be lost when implementing a shallow network, such as the Xception model. Experimental results corroborated the argument. In this instance as well, inception modules lie in the centre of a discrete spectrum, encompassing pointwise and depthwise separable convolution layers. As a result, they optimize the classification efficiency while keeping computation costs that are equivalent to those incurred by inception-based networks by adopting depthwise separable convolutions for the typical inception modules. An illustrative structure of the Xception model is shown in Fig 3.

### DenseNet169

**Fig 4:**
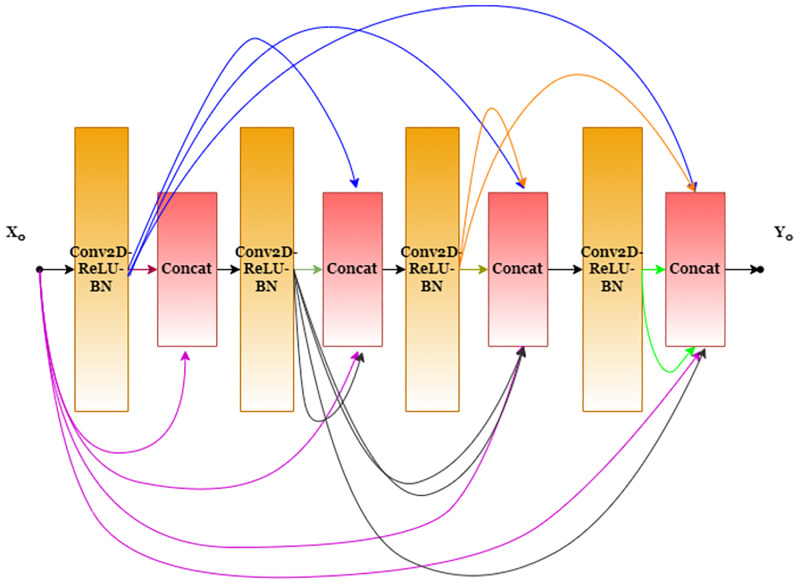
A basic block used in the DenseNet architecture. Modified from Huang et al. [31].

Earlier researchers faced a frequent problem with CNNs—when the model is deep, the derivative value calculated for backpropagation becomes low, and the gradient update becomes insignificant. It is commonly referred to as the vanishing gradient problem. To address this problem, the researchers came up with the idea of interconnecting all the other layers to maximize the flow of information. DenseNet [31] consists of seven dense blocks, where each block has 4 convolutional sublayers. The output from each of the sub-layers is concatenated into one input tensor and propagated through the subsequent sub-layers. Every sublayer is symmetrical in nature and consists of the following sequence: Batch Normalization, ReLU activation function, Dropout and Convolution. In every case, the dropout probability is 0.5, and also convolution kernel size is fixed at 5. Fig 4 shows the dense connections employed in the architecture. These are inspired by the skip connections of ResNet, where a layer receives the feature map only from the last layer. These dense connections help in producing more diversified features as each layer receives all the preceding layers’ feature maps as the input. The role of convolutional sub-masking within the DenseNet helps realize better gradient flow. The dense connections among the sub-layers follow a sequential flow. A sublayer completes its forward pass only if all previous sublayers have completed their computations. The dense connections allow for better gradient flow with fewer parameters.

### Beta normalization based ensemble scheme

Generally, for a deep learner, the generated probabilities or the confidence scores are many times higher, even for false positive scenarios. This behavior does not allow an aggregator to learn complementary information obtained from multiple base learners. To deal with this, we propose a normalization technique based on the Beta function. The Euler integral of the first kind or the Beta function as it is commonly known has a wide range of applications in calculus primarily for approximations. The Beta function is calculated as in Eq 1, where *p*, *q* ∈ ℜ^+^.

$$\begin{array}{c} \beta \left(p,q\right)=\int_{0}^{1}t^{p-1}{\left(1-t\right)}^{q-1}dt \end{array}$$ (1)

The Beta function in mathematics is regarded as generating a close association between sets of inputs and outputs by strongly associating each input value with the associated output value by changing the inputs to exhibit significant representational ability. We considered utilizing the Beta function in the proposed ensemble system since it has the potential to map inputs to coherent outputs. This is because the goal of the ensemble learning is to build an appropriate aggregation of values (here the outputs generated by the base learners), and the use of the Beta function to learn an aggregation can be substantiated for this purpose. To model this, we first consider the ordered pair (*p* + 1, *q* + 1) as (*α*, *γ*), where *α* is the observed probability and *γ* is the maximum achievable probability. Also, it should be noted that *α* will always be lesser than 1 which makes this function a monotonically decreasing function. Therefore, for proper utilization, we subtract the whole value from 1 in our case. As a result, the calculation of the normalized probability scores *β*(*p*_*n*_) is calculated as in Eq 2. The value of *γ* is 1, and *α* is the observed probability. We consider that *t* is integrated within the limits of 1 and 0, and Eq 4 refers to this integration. The final calculation is according to Eq 5.

$$\begin{array}{c} \beta \left(p_{n}\right)=1-\int_{0}^{1}t^{\alpha }{\left(1-t\right)}^{\gamma }dt \end{array}$$ (2)

$$\begin{array}{c} \beta \left(p_{n}\right)=1-\int_{0}^{1}\left(t^{\alpha }-t^{\alpha +1}\right)dt \end{array}$$ (3)

$$\begin{array}{c} \beta \left(p_{n}\right)=1-{\left[\frac{t^{\alpha +1}}{\alpha +1}-\frac{t^{\alpha +2}}{\alpha +2}\right]}_{0}^{1} \end{array}$$ (4)

$$\begin{array}{c} \beta \left(p_{n}\right)=\frac{\alpha^{2}+3\times \alpha +1}{\alpha^{2}+3\times \alpha +2} \end{array}$$ (5)

After normalizing the probabilities, let us consider $P_{j}^{*}\left(x_{i}\right)=\left(P_{j}^{I}\left(x_{i}\right),P_{j}^{X}\left(x_{i}\right),P_{j}^{D}\left(x_{i}\right)\right)$ concerning the *j*^*th*^ class label, where *P*^*I*^, *P*^*X*^, *P*^*D*^ represent the normalized probabilities for Inception, Xception and DenseNet models for the *i*^*th*^ sample. Correspondingly, the use of the sum rule for each class outputs this: $\xi_{j}\left(x_{i}\right)=P_{j}^{I}\left(x_{i}\right)+P_{j}^{X}\left(x_{i}\right)+P_{j}^{D}\left(x_{i}\right)$. The final class label ${\hat{y}}_{i}$ is assigned in accordance with Eq 6

$$\begin{array}{c} {\hat{y}}_{i}=\underset{j}{\text{arg max}}\left\{\xi_{j}\left(x_{i}\right)\right\} \end{array}$$ (6)

**Fig 5:**
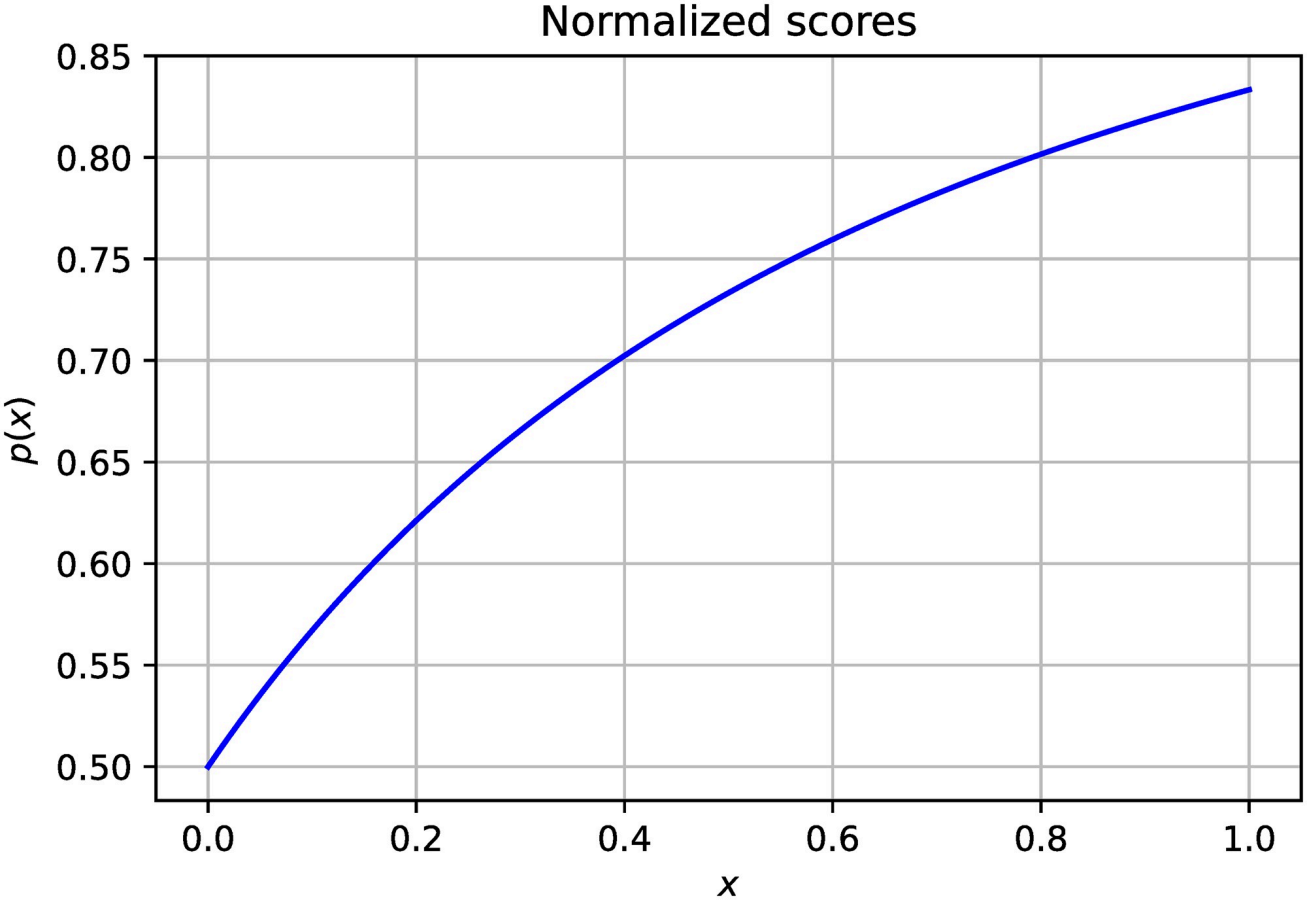
Normalized scores generated by the Beta function. The normalized values are plotted against the y-axis.

A graphical representation of normalized probabilities is shown in Fig 5. From the figure we observe that the scores are very close enough to learn an aggregation. For the convenience of the readers, we present an example in Table 1 to show how the proposed methodology works. We observe from Table 1 that the use of the rule and the Beta transformation results in some differences in the predictions. We observe that the probabilities are changed to become closer, which, in turn, learns a good aggregation after transformation. It must be noted that ∂*β*(*x*)/∂*x* > 0∀*x* ∈ (0, 1), which means that the function is monotonically increasing throughout, whereas ∂^2^*β*(*x*)/∂*x*^2^ < 0∀*x* ∈ (0, 1) means that the function’s nature is concave downward, which also means that the probabilities with higher values are relatively less important. This fact helps to reduce the gap between true positive and false positive predictions.

**Table 1:** A toy example of the Beta normalization scheme with different probability values. *P*_*M*_&*P*_*NM*_ refer to probabilities for Monkeypox and Non-Monkeypox classes respectively. Other symbols have usual meanings as described above.

$P_{j}^{*}\left(x\right)$	$P_{M}^{*}\left(x\right)$	$P_{NM}^{*}\left(x\right)$	$\beta (P_{M}^{*}\left(x\right))$	$\beta (P_{NM}^{*}\left(x\right))$
$P_{j}^{I}\left(x\right)$	0.9155	0.0845	0.82094	0.55765
$P_{j}^{X}\left(x\right)$	0.000112	0.999888	0.50008	0.83332
$P_{j}^{D}\left(x\right)$	0.61412	0.38588	0.7630	0.69757
*ξ*_*j*_(*x*)	**1.529732**	1.470268	2.08403	**2.08854**

## Statement of ethical approval

All procedures performed in studies involving human participants were in accordance with the ethical standards of the institutional and/or national research committee and with the 1964 Helsinki Declaration and its later amendments.

## Results and analysis

In this section, at first, we analyze the hyperparameters used in our experiments. We next go on to the primary findings and make an effort to analyse our results. We also compare the proposed ensemble of CNN models using Beta normalization to other popular ensemble methods.

### Evaluation metrics

The evaluation metrics used to evaluate the proposed method can be found below. First, we discuss some of the preliminaries followed by the metrics.

- True Positives (TP): It is a scenario when the obtained class label matches the ground truth label for the positive class.
- False Positives (FP): It is a scenario when the obtained class label does not match the ground truth label for the positive class.
- True Negatives (TN): It is a scenario when the obtained class label matches the ground truth label for the negative class.
- False Negatives (FN): It is a scenario when the obtained class label does not match the ground truth label for the negative class.

We use these observations to evaluate the following metrics

1. Accuracy quantifies the ratio of true predictions to the total number of samples. The accuracy is calculated according to Eq 7.
$$\begin{array}{c} Accuracy\;=\;\frac{TP+TN}{TP+TN+FP+FN} \end{array}$$ (7)
2. Precision score is the ratio of correct predictions for the positive class to the total number of samples predicted to the positive class. It is calculated according to Eq 8
$$\begin{array}{c} Precision\;=\;\frac{TP}{TP+FP} \end{array}$$ (8)
3. Recall score is the ratio of true positives to the sum of true positives and false negatives. It is calculated according to Eq 9
$$\begin{array}{c} Recall\;=\;\frac{TP}{TP+FN} \end{array}$$ (9)
4. F1 score is the harmonic mean of precision and recall. It can be calculated as in Eq 10
$$\begin{array}{c} F1\;=\;\frac{2\times Precision\times Recall}{Precision+Recall} \end{array}$$ (10)

### Hyperparameter selection

When training a deep CNN model, selecting the appropriate set of hyperparameters is a challenging task and it requires intensive analysis as they directly control the training processes. The two major important hyperparameters in any deep CNN model training are the learning rate and the batch size. The learning rate determines how much to modify the model’s weights each time in response to the predicted error. On the other hand, the batch size determines how many training examples a model should process in one go when the model is trained. While training deep neural networks, batch size influences the precision of the error estimation gradients. In our experiments, we have used three basic pre-trained CNN models. We have come to the right set of values for the hyperparameters through the popular grid-search method, where the learning rate is selected from {1*e* − 3,1*e* − 4,1*e* − 5}, and batch size from {8, 16, 32, 64}. All models have been trained for 30 epochs on the dataset under consideration. The learning rate is scheduled to the 1/10^*th*^ of its initial value after 20 or 25 epochs depending on the base model to minimize any kind of overfitting. The experiments were performed on a random 80 : 20 split of the dataset. Later, we ensemble the predictions of three base models using the sum rule after normalizing them using the proposed Beta function-based scheme.

**Fig 6:**
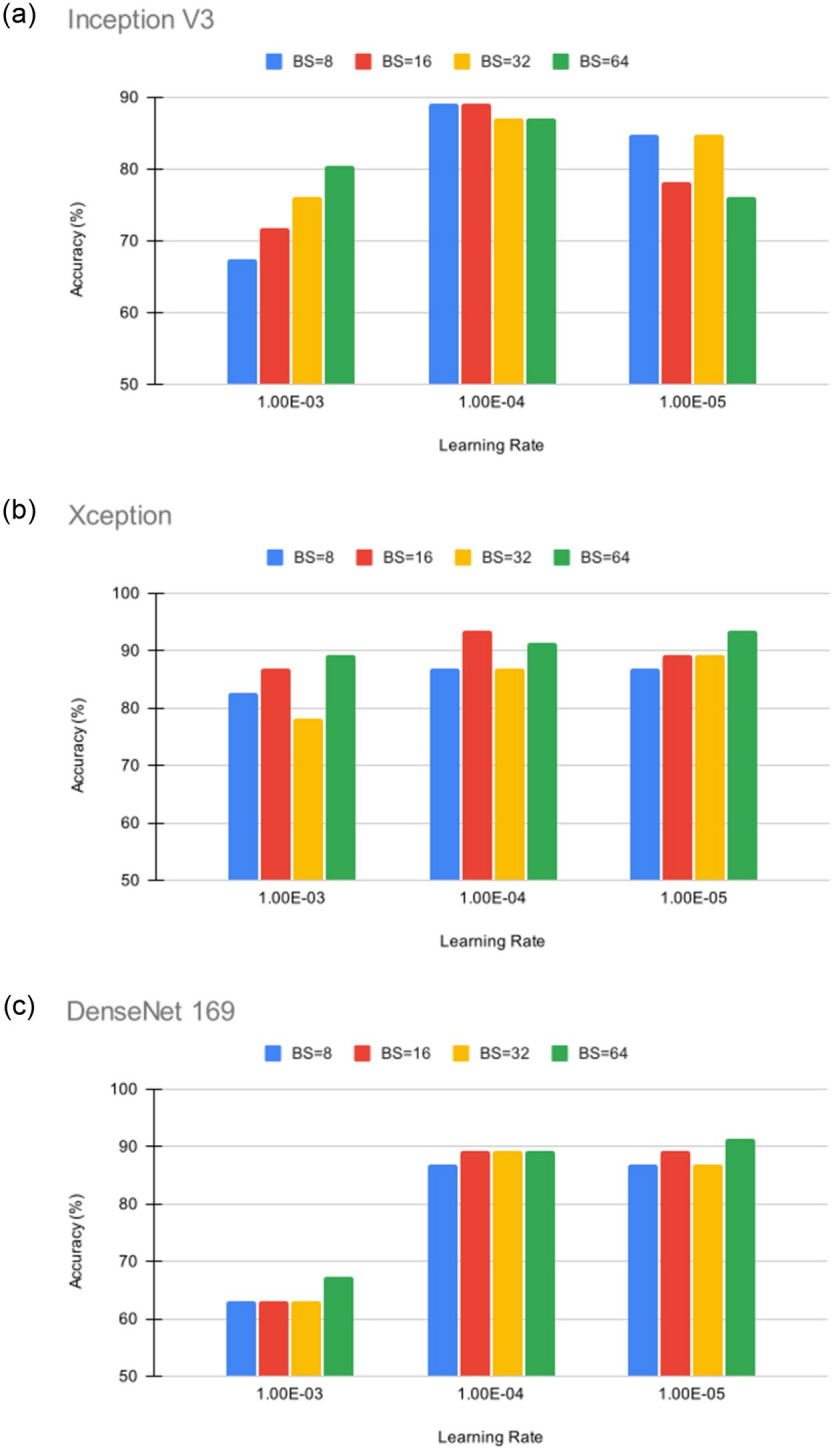
Ablation study concerning different batch sizes and learning rates on the Fold-1 of the dataset. The base models are mentioned on the top of each of the bar charts.

We have also performed experiments using the 5-fold cross-validation setting by retraining 3 pre-trained (on the ImageNet dataset) CNN models, namely Xception, InceptionV3 and DenseNet169. The result of each model on 5 folds is given in the subsequent subsections of this paper. From Fig 6 it is clear that the three models give the best results with batch size 16 and learning rate 1*e* − 4.

### Results

**Fig 7:**
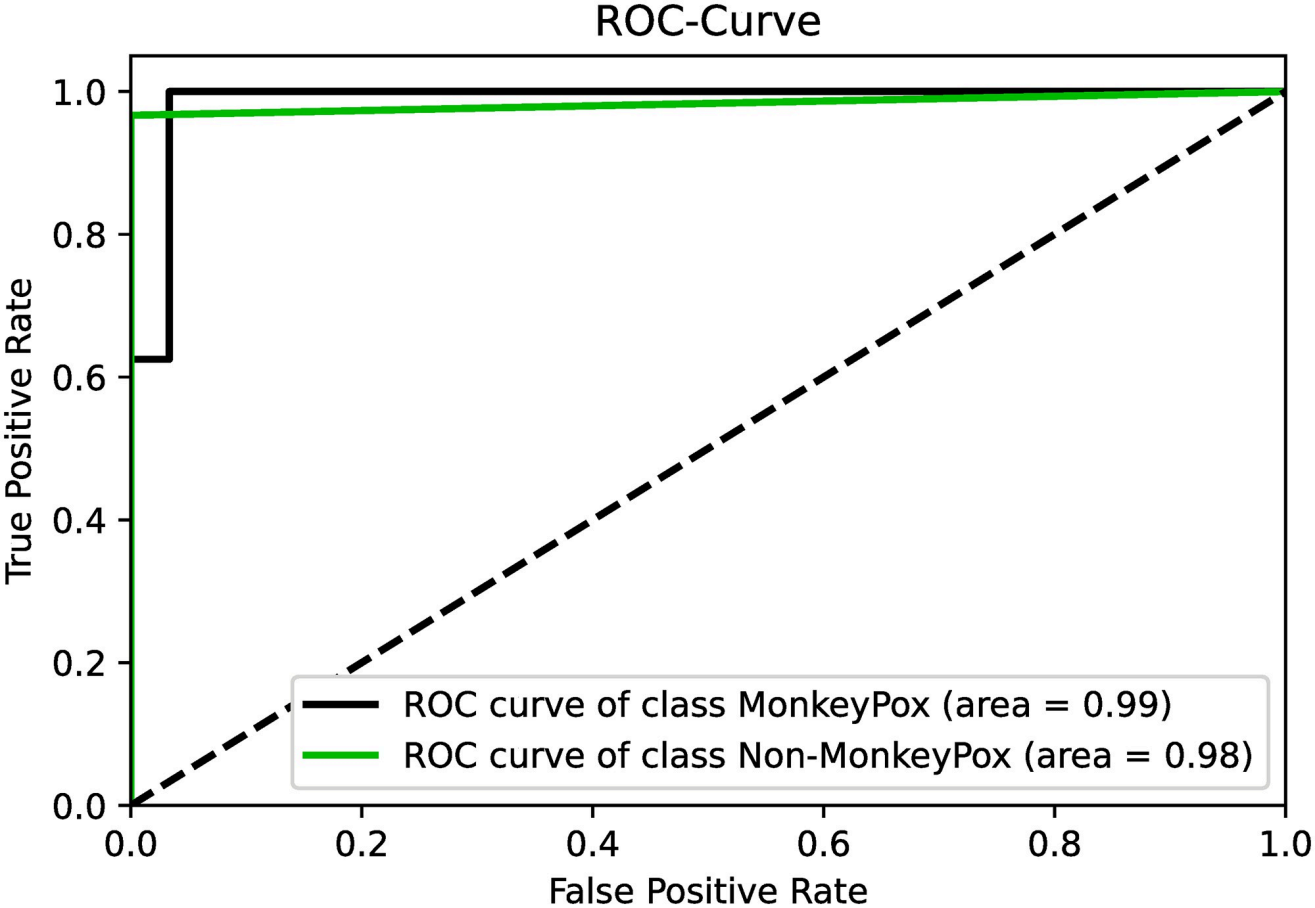
Post-ensemble ROC curves for both the Monkeypox and non-Monkeypox classes concerning Fold-1 of the experiment.

**Fig 8:**
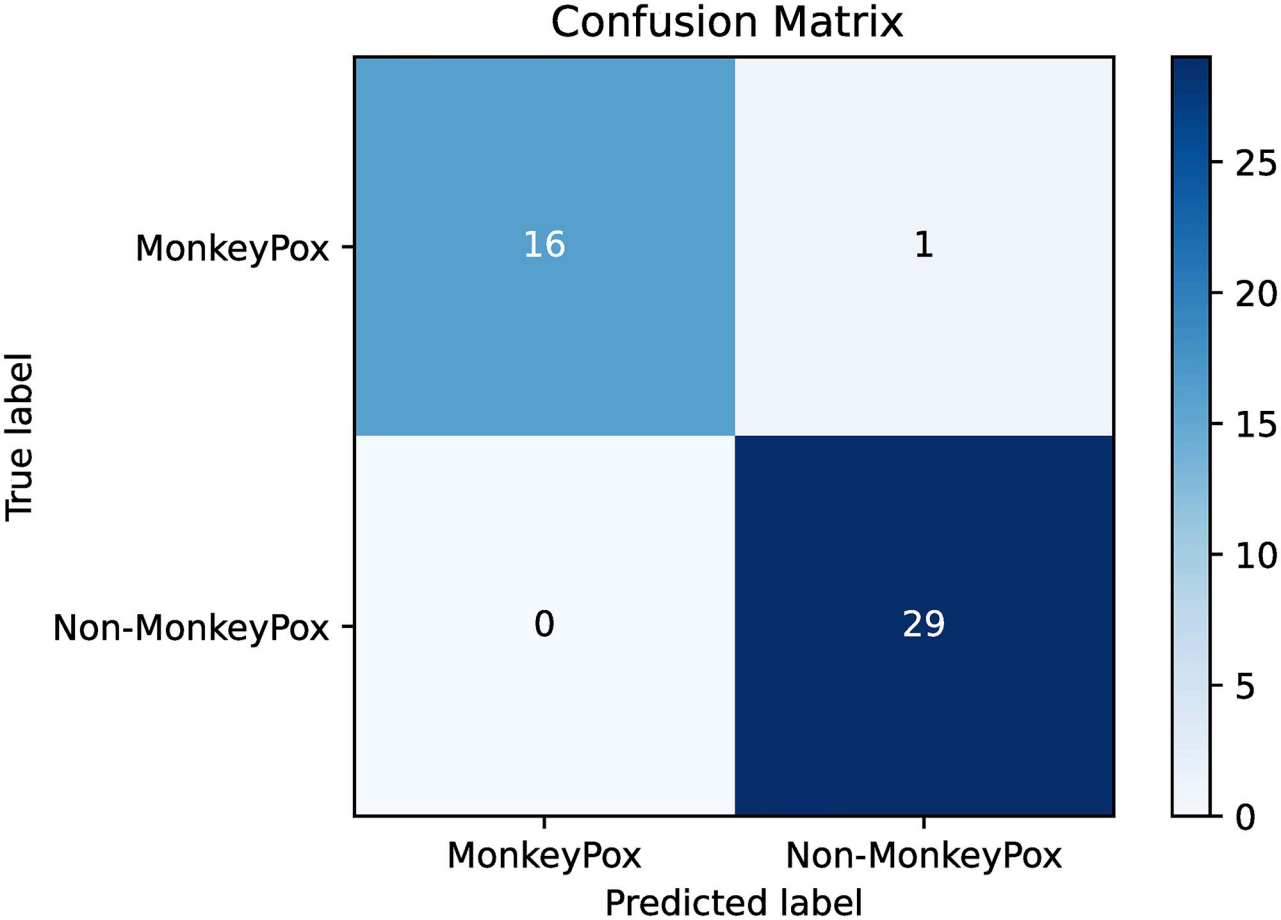
Post-ensemble confusion matrix for Fold-1 of the experiment.

As mentioned, the proposed method experiments on a binary-class Monkeypox dataset namely the Monkeypox Skin Lesion dataset. The proposed approach is evaluated using a 5-fold cross-validation setting. Table 2 shows the fold-wise accuracy on the randomly split 5-folds. It is evident from the results that, apart from the 4^th^ fold, the suggested ensemble technique significantly improves the accuracy compared to the accuracy of the best base model for the corresponding fold. On average, this method has given an overall boost of 2.17% on the five folds. This boost can be attributed to the enhancement scheme’s successful acquisition of possible complementary information obtained from base classifiers’ confidence scores. The basic classifiers’ ability to avoid overfitting is a key factor in the effectiveness of the suggested technique. When evaluating any approach, it is an important task to evaluate the performance class-wise. Taking this into account, we present the receiver operating characteristic (ROC) curve in Fig 7. The confusion matrix is also presented in Fig 8. From these figures, we can ensure that our method is able to classify each of the two classes comfortably.

**Table 2:** Performance comparison with respect to accuracy for the base learners and the proposed ensemble method on the Monkeypox skin lesion dataset using 5-fold cross-validation methodology. All values are reported in %.

Fold	Xception	InceptionV3	DenseNet169	Best Model	Ensemble	Boost
Fold 1	93.48	89.13	89.13	93.48	97.83	4.35
Fold 2	89.13	91.30	91.30	91.30	93.48	2.18
Fold 3	93.48	93.48	89.13	93.48	95.65	2.10
Fold 4	93.33	91.11	95.56	95.56	95.56	0.00
Fold 5	82.22	80.00	82.22	82.22	84.44	2.20
Average	90.33	89.45	89.47	91.21	93.39	2.17

### Comparison with state-of-the-art ensemble methods

**Fig 9:**
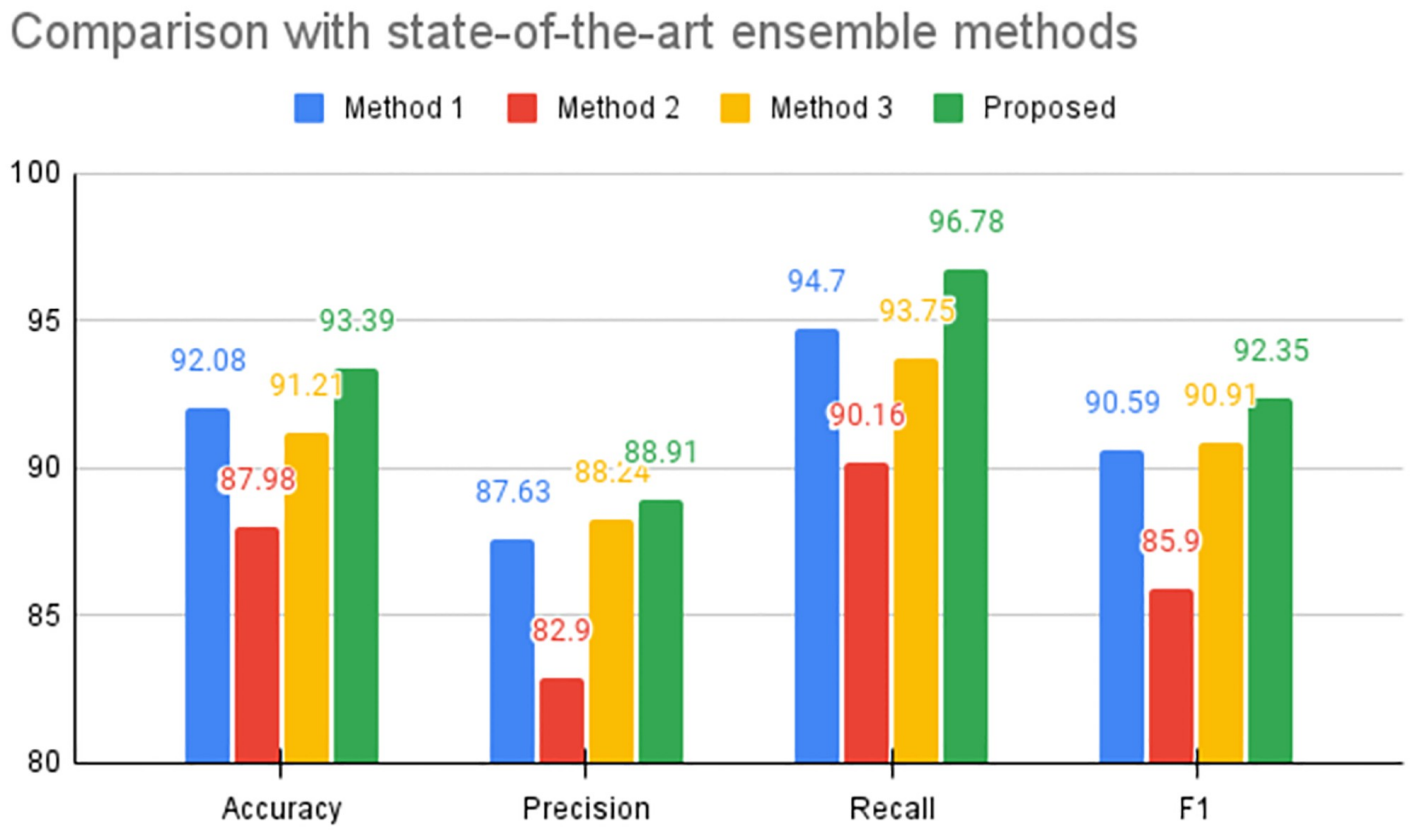
Performance comparison of the proposed method with state-of-the-art ensemble techniques. Here, Method 1 refers to the work reported in [22], Method 2 refers to the work reported in [32] and Method 3 refers to the work reported in [33]. Furthermore, the values presented are averaged across all 5 folds of the experiments. The reported scores are in (%).

As we stated earlier, in this paper, we present an ensemble learning-based framework to detect Monkeypox from skin lesion images. In this subsection, we have compared the results concerning several other state-of-the-art ensemble schemes. For this task, we have considered three diverse methods that leverage ensemble learning for final prediction. The work of Pramanik et al. [22] proposes a fuzzy distance-based ensemble scheme where the authors propose a minimization scheme based on the observed label and the ideal solution. In the work of Tabakov et al. [32], the authors use the Sugeno integral to aggregate the probability scores. Furthermore, a work by Kundu et al. [33] proposes a fuzzy ranking scheme based on the Gompertz function to aggregate the outcomes of the base deep learners. It should be noted that all of these methods are based on different strategies. As a result, these methods give us the best opportunity to compare the robustness of our method. The results are given as a bar chart under Fig 9. From this figure, it is clear that the proposed Beta function-based ensemble scheme gives the best results when compared with the state-of-the-art methods for all the metrics. We can comment that this performance enhancement becomes possible due to the normalization process proposed in this work. The normalization process learns to aggregate the probability scores effectively, thereby allowing a better composition of complementary information to be exploited. In the case of method [22], one major shortcoming is the use of cosine distance, as stated by the authors. For the method reported in [32], an intergral approximation is used, which negates the very marginal differences in probabilities. Whereas the method reported in [33] considers a ranking scheme based on the user defined top *k* classes. As a result, some of the candidate classes are filtered out at an early stage and thus ignored in the decision-making process.

### Evaluation using other metrics

To further investigate the behavior of our method, we present the comparative precision, recall, and F1 scores in Tables 3–5 respectively. These results demonstrate that our method typically outperforms the best scores or, in certain cases, maintains the greatest value. Furthermore, the dataset appears to be imbalanced given that precision values are often lower than recall scores. Additionally, there are fewer images here than in conventional datasets. We may see situations where the precision is 100%, which implies there are never any false positives. In other words, no image other than a Monkeypox image is classified as a Monkeypox image. Whereas if recall rate is 100%, there have been no instances of non-Monkeypox subjects being mistakenly diagnosed as having the disease.

**Table 3:** Fold wise precision scores concerning the base models and the ensemble method. All scores are reported in %.

Fold	Method			
	Xception	Inception V3	DenseNet 169	Ensemble
Fold 1	88.35	88.24	76.47	94.12
Fold 2	73.68	89.47	84.21	84.21
Fold 3	88.46	88.46	84.67	92.31
Fold 4	100.00	94.12	100.00	100.00
Fold 5	69.57	65.22	73.91	73.91
Average	84.01	85.10	83.85	88.91

**Table 4:** Fold wise recall scores concerning the base models and the ensemble method. All scores are reported in %.

Fold	Method			
	Xception	Inception V3	DenseNet 169	Ensemble
Fold 1	100.00	83.33	92.86	100.00
Fold 2	100.00	89.47	94.12	100.00
Fold 3	100.00	100.00	95.65	100.00
Fold 4	85.00	84.21	89.47	89.47
Fold 5	94.12	93.75	89.47	94.44
Average	95.82	90.15	92.31	96.78

**Table 5:** Fold wise F1 scores concerning the base models and the ensemble method. All scores are reported in %.

Fold	Method			
	Xception	Inception V3	DenseNet 169	Ensemble
Fold 1	90.32	85.71	83.87	96.97
Fold 2	84.85	89.47	88.89	91.43
Fold 3	93.88	93.88	89.80	96.00
Fold 4	91.89	88.89	94.44	94.44
Fold 5	80.00	76.92	80.95	82.93
Average	88.19	86.97	87.59	92.35

### Error case analysis

**Fig 10:**
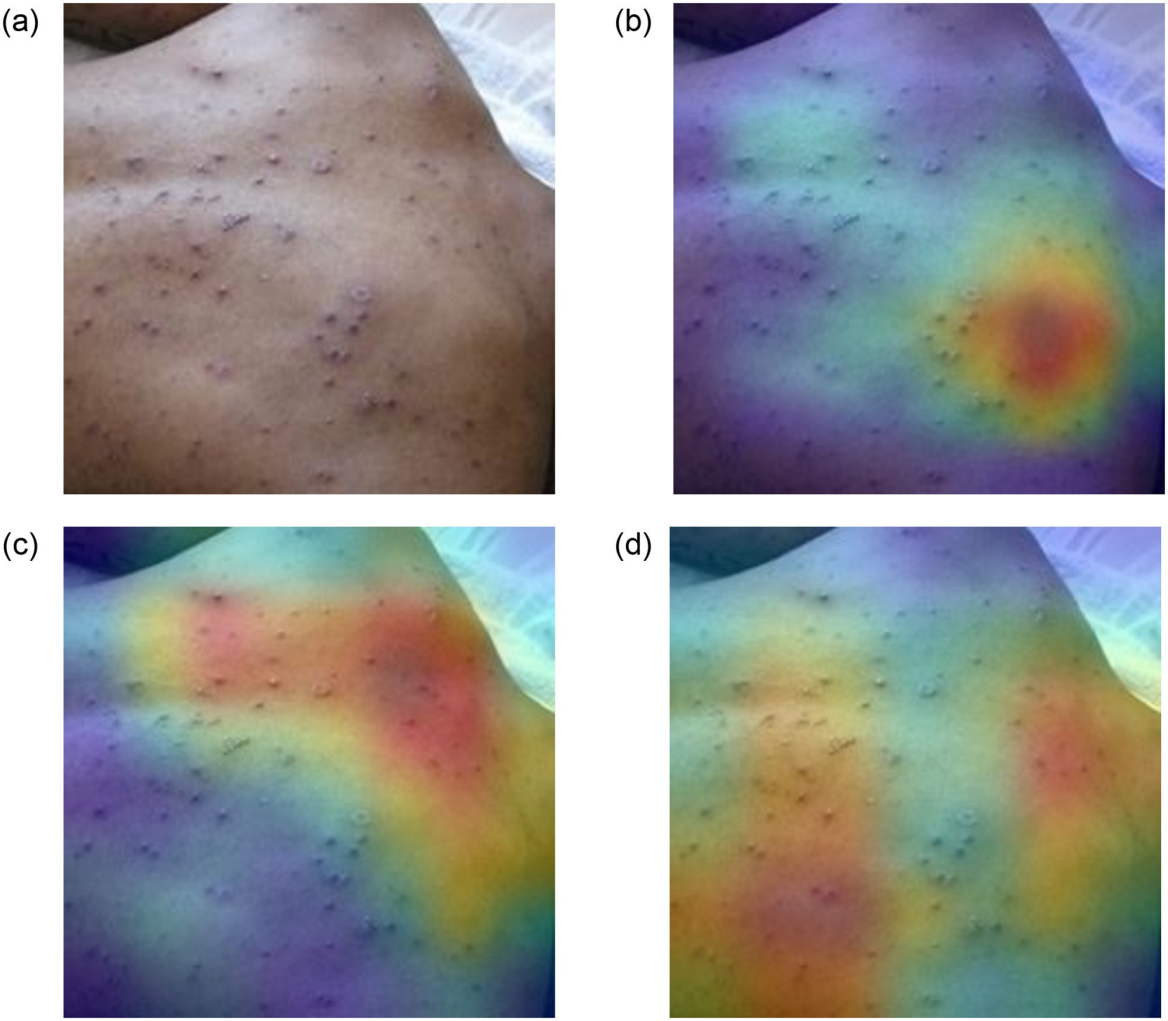
Grad-CAM for an image belonging to Non-Monkeypox class but classified as Monkeypox. The top left image is the original image followed by Grad-CAMs from the Xception (Top Right), Inception V3 (Bottom Left) and DenseNet 169 (Bottom Right) model. The probabilities of classification probabilities are as follows: Xception-0.9945 (Monkeypox), Inception V3-0.9081 (Non Monkeypox) and DenseNet 169-0.9999 (Monkeypox).

**Fig 11:**
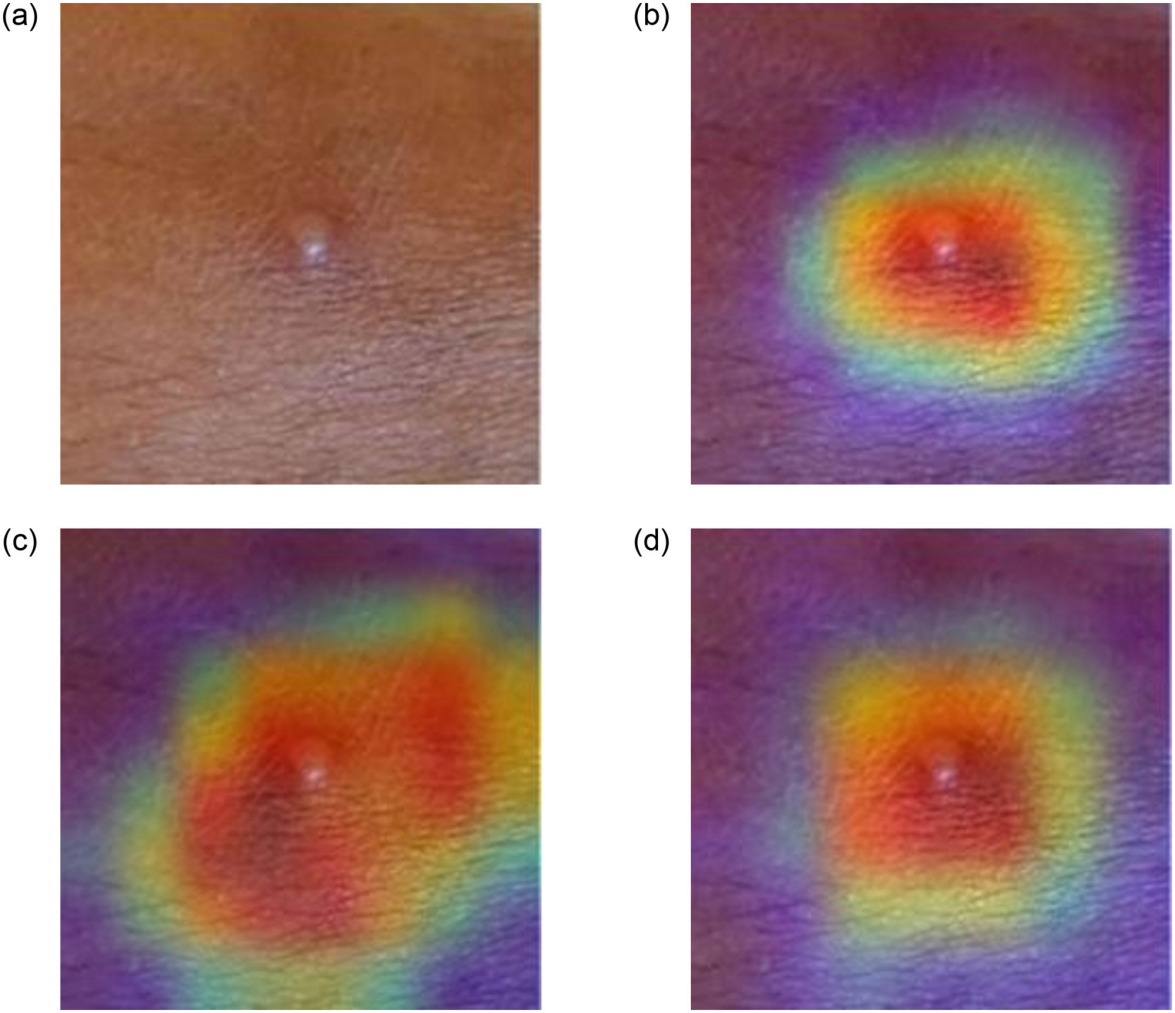
Grad-CAM for an image belonging to Monkeypox class but classified as a Non-Monkeypox class. The top left image is the original image followed by Grad-CAMs from the Xception (top right), Inception V3 (bottom left) and DenseNet 169 (bottom right) models. The classification probabilities are as follows: Xception-0.7049 (Non-Monkeypox), Inception V3-0.9947 (Non-Monkeypox) and DenseNet 169-0.9789 (Non-Monkeypox).

While proposing any method, it is always important to analyse the limitations of the proposed method. The feature extraction process is at the heart of a deep learning-based classification task. The more informative the feature, the higher the chance of an accurate classification. Gradient-weighted Class Activation Mapping (Grad-CAM) is a powerful tool that researchers are now using to simulate the feature maps generated by CNN models. We also rely on this tool as well, and in Figs 10 and 11, we show the Grad-CAM for a test sample of a skin lesion image to visually understand the feature maps generated by deep learners. The Grad-CAM images are generated using the outputs of the architectures’ final convolutional layer. In Fig 10 we present an example where the skin lesion is of Non-Monkeypox type but is classified as Monkeypox type. On the other hand, in Fig 11 we present an example where the skin lesion is of Monkeypox type but is classified as Non-Monkeypox type.

It should be noted that in Fig 10 each of the models focuses on different regions with overlap in the bottom region. The main aim of an ensemble learning framework is to form an association of complementary features. This seems to be the case here. It is important to note that the lesions are spread out, and as a result, it becomes difficult for the models to focus on a single region.

In Fig 11, we observe a skin lesion that is more present locally compared to the precious figure. Although the lesion does not appear mature enough to be classified into any of the categories, this may be a strong reason why Grad-CAMs suggest the area of interest to be more spread out compared to the size of the lesion. Like in the case of the Xception model, the region of interest is much more localised around the lesion area, consequently, the probability obtained is the lowest of all. However, the clean surface, combined with the small lesion size, leads the deep models to incorrectly classify the image as a non-Monkeypox image.

## Conclusion

The recent outbreak of Monkeypox and its harmful impacts pose a vulnerable challenge to society. Early diagnosis along with treatment with the best possible medical advice is the only way to deal with this disease. In this paper, we present an ensemble learning-based framework comprising three deep learners as the base models. We propose a Beta function-based normalization scheme for probability normalization followed by the sum rule-based ensemble. We test the method on a publicly available Monkeypox skin lesion dataset using a 5-fold cross-validation methodology to show the robustness of the proposed method.

One major limitation of working on this research topic is the lack of datasets. Hence, we want to augment the dataset size using some latest deep learning-based models. We also want to work on attention-based methods to highlight important regions for better diagnosis. Since our method provides an end-to-end solution, it may be considered for real-time deployment. However, the advice of medical professionals should be carefully considered before taking any such steps. Additionally, we may collaborate with medical professionals to get handcrafted features to increase the robustness of this method.
